# Evaluation of Gelatin/Hyaluronic Acid-Generated Bridging in a 3D-Printed Titanium Cage for Bone Regeneration

**DOI:** 10.3390/jfb14120562

**Published:** 2023-11-30

**Authors:** Seong-Su Park, Ume Farwa, Mosharraf Hossain, Soobin Im, Byong-Taek Lee

**Affiliations:** 1 Department of Regenerative Medicine, College of Medicine, Soonchunhyang University Cheonan, Cheonan 31151, Republic of Korea; sspark@sch.ac.kr; 2 Institute of Tissue Regeneration, Soonchunhyang University Cheonan, Cheonan 31151, Republic of Korea; farwa@sch.ac.kr (U.F.); isbrzw@schmc.ac.kr (S.I.); 3 Department of Neurosurgery, Soonchunhyang University, Bucheon Hospital, Bucheon 14584, Republic of Korea; mosharraf121134@sch.ac.kr

**Keywords:** gelatin, hyaluronic acid, in vivo and in vitro analysis, titanium cage, 3D printing, bone regeneration

## Abstract

3D-printed titanium (Ti) cages present an attractive alternative for addressing issues related to osteoporosis-induced fractures, accidental fractures, and spinal fusion surgery due to disc herniation. These Ti-based bone implants possess superior strength compared to other metals, allowing for versatile applications in orthopedic scenarios. However, when used as standalone solutions, certain considerations may arise, such as interaction with soft tissues. Therefore, to overcome these issues, the combination with hydrogel has been considered. In this study, to impart Ti with regenerative abilities a 3D-printed Ti cage was loaded with gelatin and hyaluronic acid (G-H) to improve the cell attachment ability of the Ti-based bone implants. The void spaces within the mesh structure of the 3D Ti cage were filled with G-H, creating a network of micro-sized pores. The filled G-H acted as the bridge for the cells to migrate toward the large inner pores of the 3D Ti cage. Due to the microporous surface and slow release of gelatin and hyaluronic acid, the biocompatibility of the coated Ti cage was increased with an elevation in osteoconduction as depicted by the up-regulation of bone-related gene expressions. The in vivo implantation in the rabbit femur model showed enhanced bone regeneration due to the coated G-H on the Ti cage compared to the pristine hollow Ti cage. The G-H filled the large holes of the 3D Ti cage that acted as a bridge for the cells to travel inside the implant and aided in the fast regeneration of bone.

## 1. Introduction

Human bone has multiple functions with hierarchical structural adoptions to perform variable functions [1]. The two main structures of the bone are the cancellous bone and the outer cortical bone [2]. The dynamic remodeling process helps heal the bone and adapt to the mechanical loads by absorbing and replacing the old micro-cracked bone. Despite the regenerative properties, bone defects and injuries due to trauma can cause severe diseases (osteoporosis, osteopaenia, and pagets) and nonunion of bone. Dealing with the aging society and maintaining the patient’s quality and activities of life have led to the utilization of orthopedic implants.

Bone fixation and implant materials made from various metals like stainless steel, cobalt, and machined titanium alloy have been extensively used. However, these materials often encounter issues such high elastic modulus as compared to the bone. To address these challenges, there is a growing trend in the use of 3D-printed Ti cages that overcomes the problem of elastic modulus. This is particularly noteworthy for non-biodegradable graft materials [3].

The Ti alloy’s excellent mechanical properties and safety have rendered it an ideal candidate for orthopedic implants. With the advent of 3D printing technology for titanium alloy, implants can be tailored to meet individual needs. One of the popular techniques for 3D-printed Ti implants is selective laser melting (SLM) [4,5]. Additionally, direct metal laser sintering (DMLS) has been recently researched for higher resolution and the use of a low-temperature laser compared to SLM, contributing to the successful manufacturing of Ti implants [6]. The 3D printing of the Ti-implants has several advantages, such as manufacturing cycle, personalized customization, and cost-effectiveness. Nevertheless, these bone implants face many challenges which need to be addressed. One of the challenges is the bio-inertness of titanium-based alloys [7]. The 3D implants have large holes that are difficult to fill by the cells, thereby displaying delayed bone regeneration.

The source of gelatin (G) is collagen (the most abundant component of ECM) obtained from the bones and skin of animals by chemical and thermal treatment. Therefore, the chemical structure of gelatin is closely related to its precursor collagen [8]. Gelatin has a linear structure consisting of glycine-X-Y amino acid repeated units. Gelatin retains the RGD (Arg-Gly-Asp) sequence [9]. RGD is known to help in cell attachment, repairment, integration, and proliferation. RDG, being considered a biomimetic peptide, can accelerate tissue regeneration and protect cells from apoptosis [10,11]. Due to the biochemical resemblance of gelatin to collagen, it is degraded by protease enzymes such as metalloproteases and collagenase, thereby rendering it non-toxic to the body [12,13].

Hyaluronic acid (H), distributed throughout the human body, is a significant component of ECM contributing to the hydration and stability of cells. As a natural polysaccharide, it offers high biocompatibility and helps in the proliferation of cells. It is constituted of two units, D-glucuronic acid and N-acetyl glucosamine disaccharide, and due to the presence of hydroxyl groups, it is hydrophilic. It can be rendered as a cross-linked hybrid hydrogel with proteins and covalently attach to metallic surfaces for coating purposes [14]. Through CD44 surface receptor signaling, it is known to promote cell proliferation and movement [15]. Depending on the molecular weight of H, the physicochemical, biological, and physical properties also vary. H with molecular weight 20–200 kDa displays embryonic development and healing abilities [16]. In contrast, 800 kDa to 1900 kDa H helps in osteogenic differentiation and new bone formation [17,18].

A combination of G-H has been used to prepare a scaffold for bone regeneration in combination with various biomaterials showing remarkable biological activities. G-H offers a unique physical and biological feature that elevates the innate ability of the body to regenerate cells. Especially they coordinate cell migration, proliferation, and adhesion for bone regeneration [19,20]. The ability of G-H to help in cell migration makes them a reliable material to fill the holes in the metal-based scaffold. It acts as a bridging material in assisting cells to penetrate and proliferate through the large holes, which, if left unfilled, may result in late bone formation.

There are many reports on ways to increase the biocompatibility of Ti-based implants by coating, chemical modification, and loading of the bioactive materials [21,22,23]. Although loading bioactive materials helps elevate the titanium-based scaffolds’ biocompatibility, the 3D-printed Ti scaffolds still have holes. For the cell penetration in the 3D-printed Ti scaffold, components that not only increase the scaffold’s biocompatibility but also act as a bridge that helps the cell migrate toward the holes for new bone formation are needed.

In this study, we have developed a 3D-printed Ti cage loaded with G-H. G-H loading can fill the vacant holes of the scaffold, which can increase biocompatibility and cell penetration. We hypothesized that by loading G-H to a 3D-printed Ti cage, the scaffold’s biocompatibility would be increased, and vacant holes of the scaffold would be filled, which would help better cell penetration. It was hypothesized that G-H would act as a bridging component for the cells to proliferate, resulting in early bone formation compared to the empty scaffold with delayed bone regeneration as cells cannot easily migrate to the large vacant holes. We evaluated our hypothesis based on the in vitro biocompatibility tests and in vivo implantation in the rabbit model for one and two months. The results supported our view that the G-H-bridged Ti cage shows higher bone formation than the hollow Ti cage.

## 2. Materials and Methods

### 2.1. Materials

Ti-6Al-4V ELI alloy powder (ASTM standard, grade 23) was used to fabricate the 3D- printed samples in this study. Gelatin and hyaluronic acid (mol. wt. ~1.5–1.8 × 10^6^ Da) was acquired from Sigma-Aldrich, St. Louis, MO, USA. MC3T3-E1 cells were obtained from ATCC (pre-osteoblast ATCC, CRL-2593, the American Type Culture Collection, Manassas, VA, USA). α-MEM minimum essential medium and MTT [3-(4,5-dimethylthiazol-2-yl)-2,5 diphenyltetrazolium bromide] solution were acquired from Gibco, New York, NY, USA. PS used was obtained from Bio-Whittaker, Walkersville, MD, USA. 1% penicillin–streptomycin, 10% fetal bovine serum FBS Bovine serum albumin, PBS, fluorescein isothiocyanate (FITC) conjugated phalloidin solution, Hoechst, and Methyl-methacrylate resin were obtained from Sigma Aldrich, St. Louis, MO, USA. DMSO (dimethyl sulfoxide) was obtained from Samchun Chemical, Pyeongtaek, Republic of Korea. Vinculin antibody was acquired from Millipore, Burlington, MA, USA.

### 2.2. Sample Preparation

A direct metal laser sintering 3D printer (EOSINT M280, EOS, Krailling, Germany) was used to fabricate a 3D-printed Ti alloy (Ti6Al4V) cage. The dimensions of the cage were 6 mm × 5 mm. Gelatin (Sigma Aldrich, Burlington, MA, USA) 10% (*w*/*v*) was dissolved in deionized water. Typically, 0.5% (*w*/*v*) of hyaluronic acid (mol. wt. ~1.5–1.8 × 10^6^ Da, Sigma Aldrich, Burlington, MA, USA) was added to the gelatin mixture (volume ratio 15:85). To load G and H, a 3D-printed scaffold was permeated with the mixture. The scaffold was then placed in a freezer at −80 °C and freeze-dried for 24 h.

### 2.3. Surface Morphology

In preparation for scanning electron microscope (SEM) observation, the samples were pre-processed, involving platinum (Pt) coating under vacuum conditions. To analyze the surface morphology, a scanning electron microscope (JSM-7401F, Tokyo, Japan) equipped with an energy-dispersive X-Ray spectroscope (EDS) was used. Pore diameter and surface area were measured by Autosorb iQ Station 2. An X-ray Photoelectron Spectrometer (Thermo Scientific, Waltham, MA, USA) was used to obtain the XPS data. The scanning range was 0–1350 eV with an AlKα radiation source.

### 2.4. Mechanical Properties

To evaluate the mechanical properties, a universal testing machine (Shimadzu Corporation (UH-F1000kNX, Kyoto, Japan) was used (diameter = 6 mm, height = 5 mm, *n* = 3). The deformation rate during compression was 0.5 mm/min.

### 2.5. In Vitro Biocompatibility

The scaffold’s cell proliferation and bio-availability were tested by employing MC3T3-E1 cells. A humidified incubator at 37 °C was used to maintain the cells with 5% CO_2_. A cell media that contained α-MEM minimum essential medium, 1% penicillin–streptomycin, 10% fetal bovine serum FBS, and PS was used.

Typically, 1 × 10^4^ MC3T3-E1 cells/mL were directly seeded onto each scaffold to evaluate the cell proliferation ability of the cells. Samples were sterilized before use, and a 24-well plate was used. A time frame of 1, 3, and 7 days was employed to compare cell proliferation. For visualization of the cells, cells were washed with PBS thrice at the predetermined time intervals. Fixation was performed with 4% paraformaldehyde for 10 min. For permeabilization, 0.5% Triton X-100 (Sigma Aldrich) was used for 10 min. Bovine serum albumin (BSA Sigma-Aldrich) was used as a blocking agent for 1 hr at ambient temperature. For immunostaining, fluorescein isothiocyanate (FITC) conjugated phalloidin solution was used at a concentration of 25 μg/mL with an incubation time of 12 h at 4 °C. Hoechst (Sigma Aldrich) was used for nuclei staining. A confocal microscope (Olympus, FV10i-W, Center Valley, PA, USA) equipped with FV10i-ASW2.0 software was used to visualize the cells.

For cell viability assay, cell-seeded samples were removed from the incubator at the predetermined time, and 200 μL MTT [3-(4,5-dimethylthiazol-2-yl)-2,5 diphenyltetrazolium bromide] solution (Gibco) was added, followed by incubation at 37 °C for 4 h. After addition of DMSO (dimethyl sulfoxide, Samchun Chemical, Pyeongtaek, Republic of Korea), samples were incubated for 1 hr. An ELISA reader (EL 312, Biokinetics reader, Bio-Tek instrument, Winooski, VT, USA) was used to determine the optical density at 595 nm.

Cell adhesion behavior was evaluated by seeding 1 × 10^5^ cells on the scaffold and incubated for 24 h at 37 °C. Up till BSA blocking, the same protocol was used as in the cell proliferation section. Vinculin antibody (Millipore) was used for immunostaining overnight at 4 °C, followed by phalloidin conjugated FITC (1 h) and Hoechst (5 min) staining. A confocal microscope was used to visualize the samples.

### 2.6. Cell Migration

The cell migration behavior in vitro was evaluated in the presence of G-H by seeding MC3T3E1 cells at a density of 10^4^ cells per well in a 6-well plate. The control was an empty well; other wells were coated with G-H gel. The confluent monolayer was established by incubating the well plates with cells. A 1 mL pipette tip was used to scratch the monolayer mechanically. PBS was gently used to wash the cell debris thrice. After the scratch formation, the width between the edges of the defect was measured immediately. Cells were then incubated for 24 h, and the distance was measured again. An inverted IX91 Olympus microscope was used to document the migration photographically. Three different points were used to calculate the average gap.

### 2.7. RNA Expression

The cells (MC3T3-E1) were seeded on the samples using the osteogenic media [24]. Afterward, RNA was extracted according to the manufacturer’s protocol (ReboEX, Geneall, Seoul, Korea and Hybrid R, Geneall, Seoul, Republic of Korea). To determine the total concentration, the RNA nanodrop (Thermo Fisher Scientific, Waltham, MA, USA) was employed. RNA was converted to cDNA by Maxime RT PreMix kit (LiliF, Seongnam, Republic of Korea). The gene expression was determined using a StepOneTM RealTime PCR System (Thermo Fisher, Waltham, MA, USA) equipped with PowerUp™ SYBR™ Green Master Mix (Applied Biosystems, Woburn, MA, USA). The expression of gene markers OCN (Osteocalcin), ALP (Alkaline phosphatase), COL1 (collagen 1), and Runx2 was determined. Table 1 shows the sequence of the primers.

### 2.8. In Vivo Biocompatibility

The in vivo experiment was conducted using New Zealand white rabbits (12 weeks, ~2.5 kg). Before the animal experiment was conducted, approval was obtained from the Soonchunhyang University Institutional Animal Care and Use Committee (Approval number: SCH22-0120, approval date 5 October 2022). Isoflurane (Piramal Critical Care Inc., Bethlehem, PA, USA) was utilized to anesthetize the animals. The right leg of the rabbit was shaved to make an incision. For sterilization of the site, 70% ethanol was used, followed by a povidone–iodine solution. A hole was made using a trephine drill, and the samples were placed at the defect site, followed by suturing. CO_2_ inhalation was used to sacrifice the animals after 1 and 2 months. The implanted site was harvested. Typically, 10% buffered formalin was used to fix the samples.

### 2.9. Micro-CT Analysis

Micro-CT was conducted using a SkyScan 1172 (Bruker, Billerica, MA, USA) scanner. The scanned data were constructed using the software NRecon. CTAn (Bruker, Billerica, MA, USA) software was used to investigate bone formation.

### 2.10. Histological Analysis

Methyl-methacrylate resin (Sigma Aldrich) was used to fix the samples after dehydration. A diamond abrasive cutter was used to cut the samples, followed by polishing at a thickness of 40 μm. Hematoxylin, eosin, and Masson’s Goldner trichrome staining were used. An BX53 (Olympus, Japan, Tokyo), equipped with a DP72 digital camera, was utilized to capture images.

### 2.11. Statistical Analysis

All the experiments were conducted in triplicate (n = 3 unless mentioned otherwise). All the statistical analyses were performed using GraphPad Prism version 8.0 (GraphPad Software Inc., San Diego, CA, USA). T-test and ANOVA two variance analysis were used to compare different groups. For confidence level, *p* < 0.05 was considered.

## 3. Results

### 3.1. Morphologies and Micro-Structure Analysis

**Figure 1:**
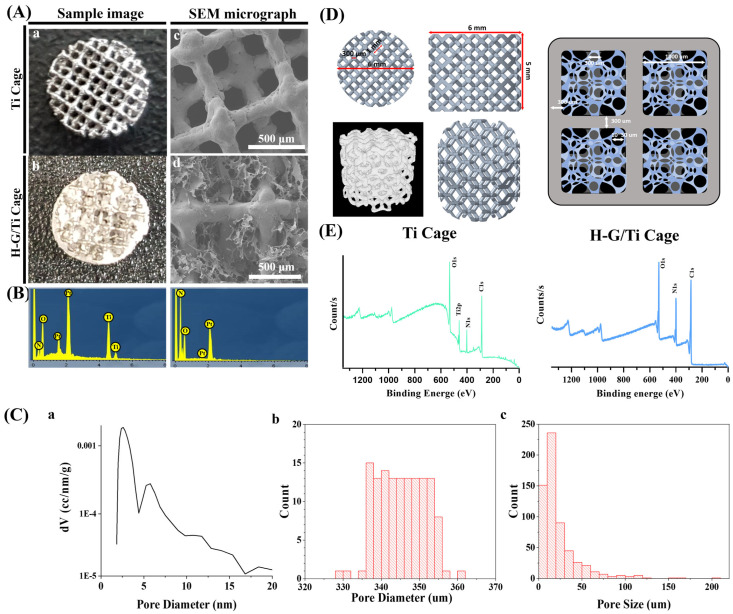
The image of Ti 3D-printed cage sample and H-G-loaded sample (**Aa**,**Ab**). SEM observation of Ti (**Ac**) and H-G-loaded Ti cage (**Ad**). EDS profile of Ti and H-G-loaded Ti cage (**B**). Pore size distribution of Ti 3D-printed cage (**Ca**). Nano-size pore distribution of the Ti structure was measured by BET(**Ca**), and micro-size pore distribution of the Ti cage and H-G/Ti cage was measured by Image J (**Cb**,**Cc**). Graphical presentation of the pore size of Ti cage and H-G-loaded Ti cage (**D**). The pore geometry of Ti cage and H-G-loaded Ti cage. XPS analysis (**E**).

A Ti 3D-printed cage harboring a lattice pattern was prepared. The Ti cage was loaded with G-H. The optical and SEM images along with EDS data showed that after loading of G-H, the holes had disappeared (Figure 1A,B). The SEM analysis showed that G-H formed a network of small pores measuring ≈10–50 μm. The lattice pattern had 1000 μm large and 350 μm small square-shaped holes (Figure 1C,D). With the loading of G-H, the surface area of the Ti cage was increased from 1.957 m^2^/g to 2.218 m^2^/g. The XPS and EDS investigation showed that the Ti cage had major peaks for Ti, O, and C (Figure 1D,E). The XPS major peaks for the G-H-loaded samples were for O, C, and N. 

### 3.2. Mechanical Properties

**Figure 2:**
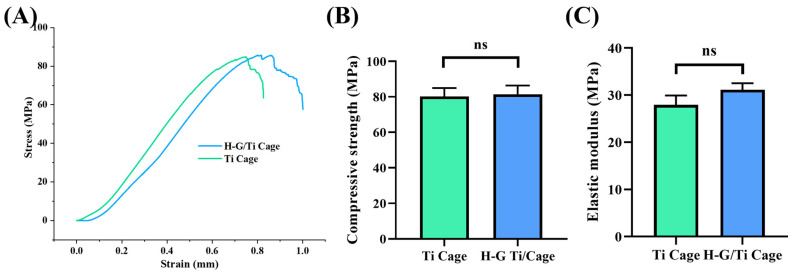
Stress–strain curve (**A**), elastic modulus (**B**), and compression strength (**C**) of Ti and H-G-loaded Ti cage, (ns: non-significance).

The mechanical properties were analyzed, focusing on compressive strength and elastic modulus (Figure 2). The compressive strength exhibited no significant difference between the Ti cage (80.1 ± 4.84) and the G-H Ti cage (81.43 ± 4.97). Although the elastic modulus was higher in the G-H Ti cage (31.14 ± 1.36) compared to the Ti cage (27.96 ± 1.97), the difference was not statistically significant.

### 3.3. Cytocompatibility

**Figure 3:**
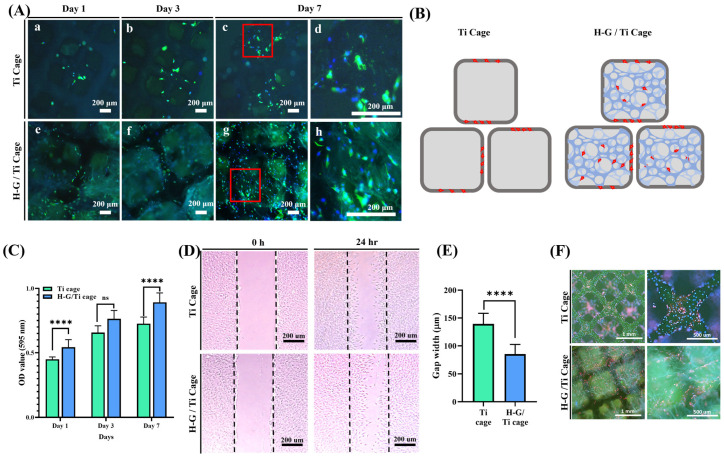
Cell proliferation (**A**), graphical representation of cell proliferation and attachment of Ti and H-G-loaded Ti cage (**B**), cytotoxicity analysis by MTT (**C**), cell migration (**D**), quantification of gap closing (**E**), and immunofluorescence staining of vinculin for cell attachment (**F**). (ns: non-significance, **** (*p* < 0.0001), The red box indicates the enlarged section on the right).

MC3T3-E1 cells were seeded on the Ti 3D cage and G-H-loaded Ti cage (Figure 3A). The cell proliferation analysis showed that cells proliferated well on the G-H-loaded scaffold compared to bare titanium. An interconnected cytoskeletal structure was observed for G-H-loaded titanium. In contrast, bare Ti displayed only cells on the edges of the square holes. In the case of the bare scaffold, the square holes were significant to be filled by the cells (Figure 3B).

The cell viability was analyzed by the MTT assay (Figure 3C). The results showed that the biocompatibility of the G-H-loaded Ti cage was higher than that of the bare Ti scaffold.

Cell migration assay was conducted to analyze the hypothesis that cells proliferated well in the presence of G-H (Figure 3D). The results demonstrated that cells migrated more when exposed to G-H gel than in its absence. The gap of the G-H gel scratch was found to be 85.43 μm, whereas, in its absence, the gap was 139.42 μm (Figure 3E).

Vinculin immunofluorescence staining demonstrated that cells adhered more to the G-H-loaded cage than to the bare Ti cage (Figure 3F). In the case of the Ti cage, the cells were attached to the edges of the scaffolds. In contrast, in the case of the G-H, cells were seen attached on the edges and in the middle of the scaffold. The bridging effect offered by loading of G-H was very evident.

### 3.4. Osteogenic Differentiation

**Figure 4:**
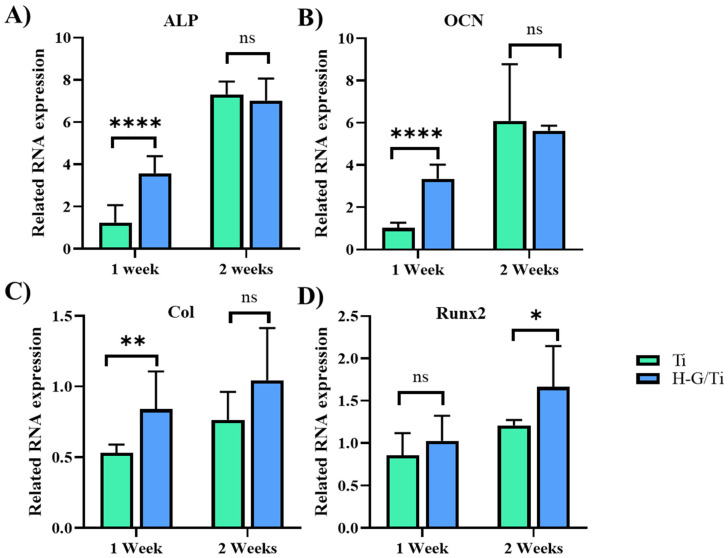
Related gene expression of ALP (**A**), OCN (**B**), Col (**C**), and Runx2 (**D**) on Ti and H-G-loaded Ti cages. (ns indicates non-significance, * (*p* < 0.05), ** (*p* < 0.01) and **** (*p* < 0.0001).

The RNA expression levels of alkaline phosphatase (ALP), osteocalcin (OCN), collagen1 (Col1), and the Runt-related transcription factor 2 (Runx2) gene were analyzed as markers for osteogenic differentiation using RT-PCR (Figure 4). Gene expression of ALP and OCN showed a significantly higher expression in the G-H-loaded cage during the first week, but no significant difference was observed during the second week (Figure 4A,B). In the case of Col, a significant increase in expression was observed in the G-H-loaded cage during the first week, with a similar level of increase noted during the second week, although no significant difference was confirmed (Figure 4C). As for Runx2, there was no significant difference during the first week, but an increase in expression was observed in the G-H-loaded cage during the second week (Figure 4D).

### 3.5. Micro-CT Analysis

**Figure 5:**
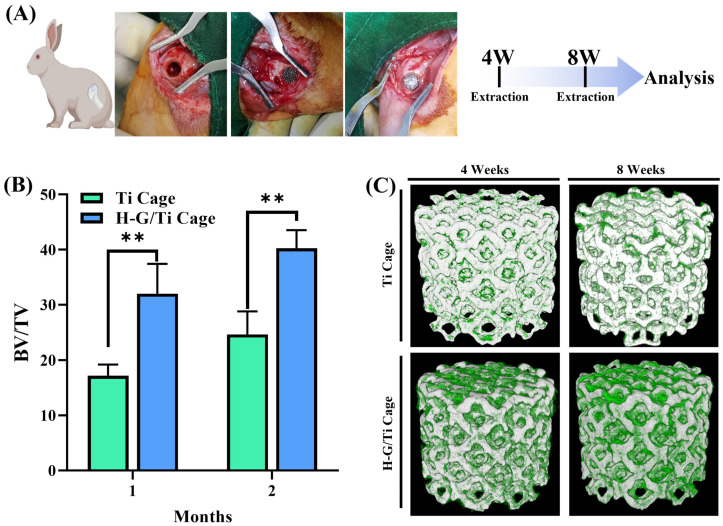
In vivo implantation in a rabbit model (**A**). Percentage of bone volume vs. tissue volume as measured by micro-CT (**B**). Micro-CT 3D visualized image (**C**). (** *p* < 0.01).

An in vivo experiment was carried out using the rabbit femur model. Samples were extracted after 4 weeks and 8 weeks (Figure 5A). BV/TV values for the Ti sample were 17.18 ± 2.01 at the first month and 24.64 ± 4.17 at the second month. In contrast, for the G-H-loaded sample, values increased significantly from 32.0 ± 5.43 at the first month and 40.24 ± 3.28 at the second month, indicating a significant increase in BV/TV with H-G-loaded (Figure 5B). The micro-CT visualization showed that bone formation was more in the G-H-loaded sample than in the bare Ti sample (Figure 5C).

### 3.6. In Vivo Histological Analysis

**Figure 6:**
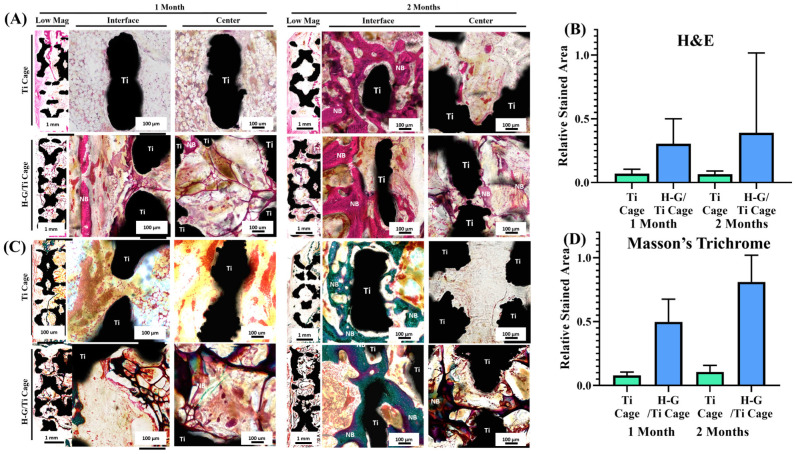
Histological evaluation of in vivo implantation for 1 and 2 months. Histological evaluation was performed by hematoxylin and eosin staining (**A**) and Masson’s Goldner trichrome staining (**C**). Relative stained areas were quantified by Image J software (**B**,**D**).

Histological analysis showed that after 4 weeks of implantation, the bare Ti cage showed only sponge-like-marrow morphology (Figure 6A,B), whereas in the case of the G-H loaded cage, the cells were attached and proliferated. G-H was also retained inside the cage along with the cells. After 2 months of implantation, bone formation occurred along the margins of the defect due to bone penetration in the case of a hollow bare Ti cage, whereas there was no bone formation in the inner zone as the square holes of the cage are hollow. In the case of the G-H cage, new bone formation was observed in the whole defect area. The inner zone was seen to have more bone formation as the cells were easily attached to the bridging network generated by the G-H bridging network. Osteoblasts were visualized in the inner zone of the G-H-loaded cage.

Masson’s Goldner trichrome staining also showed consistent results (Figure 6C,D). The bare Ti cage showed less bone regeneration, whereas the G-H cage showed more significant bone regeneration.

## 4. Discussion

3D-printed Ti cages have large holes which are difficult for cells to fill, hence delaying the process of bone regeneration. G-H loading resolves this problem by the formation of the network of pores that act as bridges for cells to penetrate and fill the big holes of the 3D-printed Ti cage. For this purpose, G-H was loaded on the Ti 3D-printed cage. Opting for hyaluronic acid, a more cost-effective yet active material to simulate the extracellular matrix (ECM), sets this study apart from others that mainly focus on loading growth factors into gelatin or incorporating materials from the calcium phosphate family for bone regeneration [25,26]. The G-H network filled the Ti cage like small bridges, making a highly interconnected web-like appearance as displayed in the SEM image (Figure 1A). The G-H scaffold not only provides the physical support to the cells to proliferate, but gelatin and hyaluronic acid are abundant in proteins such as RGD, which helps cells in the fast migration. When cells are loaded on the bare Ti scaffold and G-H-loaded Ti scaffold, the cells proliferate on the G-H scaffold, whereas in the case of the Ti scaffold, the margins are occupied. The well-spreading of cells on the G-H-loaded scaffold can be related to the increased biocompatibility of the scaffold, where the interconnected cytoskeletal network developed as the large square holes of the scaffold got interconnected by the bridging web-like network of the G-H. Based on the confocal images (Figure 3A), it can be said that cells migrated well between the square holes of the G-H-loaded Ti cage due to the bridging provided by the G-H network. Hence the bridging effect supported the fast proliferation of the cells in the inner zone of the Ti cage. Gelatin is sourced from collagen, and hyaluronic acid is a significant component of the ECM. The combined effect of G and H results in enhanced biocompatibility. Cells can proliferate and adhere in the presence of G-H. Gelatin is known to retain the RGD (Arg-Gly-Asp) sequence, which helps cells in proliferation and adherence.

In vitro analyses suggested that G-H not only increased the scaffold’s biocompatibility but also provided a rather unique network of bridges that helped cells proliferate and penetrate the square holes of the cage. In the case of the bare Ti cage, the square holes were large, hindering the cells’ fast proliferation to fill the holes, whereas after loading of G-H, the holes were filled by the network of bridges, which fastened the process of cell proliferation. It can be assumed that this mechanism of fast proliferation of the cells can also help in the fast regeneration of bone in the in vivo model.

In the comparative analysis of RNA expression levels using RT-PCR, a remarkable increase in the expression levels of ALP, OCN, and Col was observed in the G-H scaffold at the 1 week time point. This observation suggests that the G-H-loaded scaffold induces the entry of pre-osteoblasts into the process of osteoblast differentiation) [27]. Although similar expression levels were observed at 2 weeks in both scaffolds, the significantly increased expression of Runx2 in the G-H-loaded samples at this time implies a rapid differentiation of pre-osteoblasts into immature osteoblasts induced by the loading of G-H [28].

The micro-CT visualization supports the hypothesis that the G-H-loaded scaffold can help in the fast proliferation of cells, which can facilitate fast bone regeneration. Cell proliferation due to the bridging network created by G-H assisted in filling the square holes of the scaffold vastly compared to the bare Ti scaffold. In the case of the bare Ti cage, the square holes were difficult to fill, resulting in delayed bone formation.

**Figure 7:**
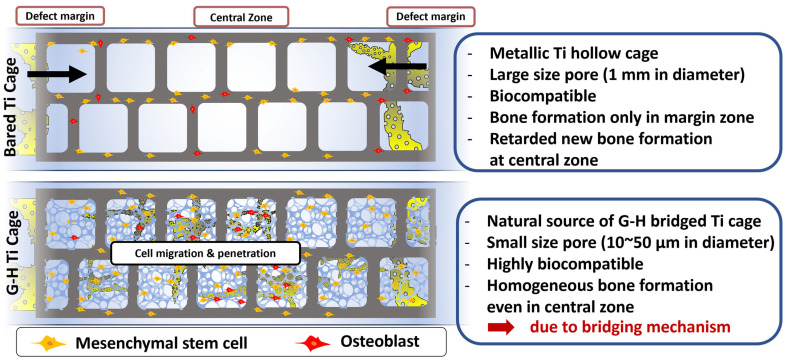
Graphical illustration representing the comparison of bone regeneration in the presence of Ti and G-H-loaded Ti cage.

Figure 7 shows the schematic summary of this work. The bare Ti cage is a hollow structure with large-sized pores. Although Ti is a biocompatible material, it did not elevate the innate bone regeneration ability. Only Ti implantation can result in bone formation along the margins of the defect, leaving the inner zone filled with sponge-like marrow. By loading natural G-H gel, a small interconnected network of pores was generated in the Ti cage resulting in the filling of the large pores. This network of pores acts as a bridge that helps the bone cells penetrate the defect’s central zone and results in homogeneous bone formation. The results support the strategy that filling the Ti cage with G-H is an alluring approach for bone regeneration.

## 5. Conclusions

A Ti 3D-printed cage was prepared and loaded with G-H. G-H loading successfully filled the holes in the Ti cage, offering a unique network of small pores. The in vitro results showed that more cells could adhere and proliferate on the loaded scaffold than on the Ti 3D-printed cage. G-H loading increased the biocompatibility and enhanced bone regeneration ability of the G-H Ti 3D-printed cage, as displayed by elevated expression of osteogenic markers for MC3T3-E1 cells. In vivo study conducted using the rabbit femur model showed that the G-H-loaded Ti 3D-printed cage resulted in higher bone formation than the bare cage. The micro-CT and histological data revealed that cells penetrated well in the G-H-loaded cage compared to the bare cage. The G-H filled the holes, and cell migration to the cage was faster than to the bare cage. The results demonstrate that employment of G-H in the Ti 3D-printed cage is a promising strategy to enhance the biocompatibility of the Ti cage and fill the holes of the scaffold, thereby generating micro-sized pores that can help cells to penetrate the scaffold, resulting in fast regeneration. In contrast to conventional cages, the G-H cage, incorporating gelatin and hyaluronic acid, establishes a favorable environment for cellular growth and differentiation. This helps bones form quickly, making the spinal fusion process faster. The innovative approach shows a promising result in reducing patient recovery periods, representing a remarkable advancement in spinal fusion surgery.

## Figures and Tables

**Table 1:** Primer sequences for real-time PCR analysis.

Gene	Sequence
mGapdh F	tctcctgcgacttcaaca
mGapdh R	ctgtagccgtattcattgtc
mCol F	tgctgcctcaaataccctttct
mCol R	tggcgtatgggatgaagtattg
mALP F	gggactggtactcggataac
mALP R	ccagttcgtattccacatc
mRunx2 F	agaagagccaggcaggtgctt
mRunx2 R	ttcgtgggttggagaagcg
mOCN F	gcttaaccctgcttgtga
mOCN R	tcctaaatagtgataccgta

F = Forward; R = Reverse.

## Data Availability

Data are contained within the article.
